# Cloning and Expression Analysis of Four Heat Shock Protein Genes in *Ericerus pela* (Homoptera: Coccidae)

**DOI:** 10.1093/jisesa/ieu032

**Published:** 2014-01-01

**Authors:** Wei-Wei Liu, Pu Yang, Xiao-Ming Chen, Dong-Li Xu, Yan-Hong Hu

**Affiliations:** Research Institute of Resources Insects, Chinese Academy of Forestry, Key laboratory of Cultivating and Utilization of Resources Insects of State Forestry Administration, Kunming 650224, China; * *These authors contributed equally to this work

**Keywords:** *Ericerus pela*, heat shock protein, heat stress, cold stress, development

## Abstract

To explore the function of small heat shock protein genes ( *shsp* s) and *hsp70* in *Ericerus pela* , we cloned the full-length cDNA sequences of *hsp21.5* , *hsp21.7* , *hsp70* , and *hsc70* and the genomic sequence of *hsc70* . Open reading frames of the four *hsp* s were 570, 564, 1,908, and 1,962 base pairs (bp), respectively, which encode proteins with calculated molecular mass of 21.5, 21.7, 69.8, and 71.6 kDa. Amino acid sequence analysis revealed the presence of the conserved Hsp motifs in all four proteins. The genomic DNA of *hsc70* had four introns. *ep-hsp21.5* was orthologous and *ep-hsp21.7* was species specific. Expression of all four transcripts during heat or cold stress and development was examined by quantitative real-time polymerase chain reaction. All four *hsp* s were upregulated during heat or cold stress in female adults, indicating a correlation between the four *hsp* s and heat or cold-stress tolerance in female adults. *ep-hsp21.7* and *ep-hsp70* were upregulated during heat stress in male larvae, implying a correlation between the two *hsp* s and heat-stress tolerance in male larvae. The four *ep-hsp* s were also upregulated during the developmental process in males, and *ep-hsp21.5* , *ep-hsp70* , and *ep-hsc70* were upregulated in females, which indicates their possible role in the developmental regulation of *E. pela* .

The Chinese white wax scale insect, *Ericerus pela* Chavannes (Homoptera: Coccidae), is a coccid insect famous for its role in wax production. The species is highly sexually dimorphic with holometamorphic males and hemimetabolic females. Females develop through the egg, first- and second-stage larva, and adult stages during their life. The female adults only have rudimentary legs and cannot move during the adult period. However, males undergo egg, first- and second-stage larva, propupa, pupa, and adult stages, emerging as winged adults. These white wax scale insects produce only one generation annually, and the females produce eggs in May (spring), which then develop through two larval stages. The insects enter the adult stage in August (summer). Female adults endure the winter and produce offsprings in the subsequent May before they die. The female adult life spans over >180 d, with a duration that lasts three seasons: summer, autumn, and winter. During its life, the female adults of *E. pela* face summer heat stress and winter cold stress. Therefore, tolerance of female adults to high and low temperatures is critical to the survival and reproduction of *E. pela* . In contrast, adult males have a life span of <1 wk, and they die just after copulation in August. In male larvae, the second instar is the longest developmental stage of 70–80 d, during which they continuously secrete large amounts of wax. During this period, heat stress and high humidity can enhance male larvae to secrete wax, forming a thick wax layer on the surface of the body ( Zhang and Liu 1997 , Chen et al. 2007a , Chen and Feng 2009 , Chen 2011 ). The larvae and the pupae stay in the wax layer until eclosion. To increase wax production, *E. pela* are usually transported to areas of high temperature and humidity to facilitate wax secretion by the male larvae. Therefore, tolerance of second-stage male larvae to high temperature stress is critically important for the wax production and male survival.

*E. pela* has been bred for over 1,000 yr, and this insect species is widely distributed in most parts of China and elsewhere, Japan, and the Korean peninsula. For wax production, high-quality females are produced in regions with an annual average temperature of 11–16°C, with the lowest temperature between −5 and −2°C, an annual rain fall of 800–1,200 mm/a, and an annual light period of 1,900–2,500 h/a. In attempts to introduce *E. pela* to various geographical regions, we found that *E. pela* was more adaptable and plastic with respect to the environment than most of other insects. It is widely distributed in the subtropical, warm temperate and temperate areas. In Changchun City of China, where the lowest temperature is below −30°C and the lowest average monthly temperature is about −13°C, *E. pela* can survive and produce offsprings. In Chongqing City, where the highest temperature is 44°C and the highest average monthly temperature is about 26°C, *E. pela* can also survive and carry on reproduction ( Zhang and Liu 1997 , Chen and Feng 2009 , Chen 2011 ). This remarkable tolerance of *E. pela* to extreme hot and cold temperatures is important for wax production. *E. pela* also provides a model to study the mechanism of extreme temperature stress adaptation in insects ( Lalouette et al. 2011 , Dunning et al. 2012 , Reiskind and Zarrabi 2012 ). Most studies on *E. pela* have focused on white wax production and related ecology ( Jiao and Zhao 1999 ; Chen et al. 2008 , 2011 ); however, the molecular mechanisms underlying the adaptability of *E. pela* to extreme temperatures have not been reported.

Heat shock proteins (Hsps) are induced by a diverse array of stresses, such as desiccation ( Lopez-Martinez et al. 2009 ), hypoxia ( Michaud et al. 2011 ), chemicals ( Planelló et al. 2011 ), metals ( Zhang and Zhang 2012 ), ultraviolet radiation ( Sang et al. 2012 ), parasitic infections ( Shim et al. 2008 ), and high population densities ( Wang et al. 2007 ). Hsps act as molecular chaperones and play critical roles in facilitating correct protein folding or refolding, preventing aggregation of denatured proteins, degrading misfolded or aggregated proteins, and transporting and assembling or disassembling proteins ( Gething and Sambrook 1992 , Sanders 1993 ). Hsps are divided into several families according to sequence homology and molecular mass, including Hsp90, Hsp70, Hsp60, Hsp40, and small Hsp (sHsp), and each family comprises one or several members. Among the Hsps, Hsp70 is well studied and is the major Hsp family that comprises both inducible and constitutive protein members. The constitutive proteins are referred to as heat shock cognate proteins (Hscs), which do not show significant change in response to stress. Proteins of the *shsp* family range in size from 12 to 42 kDa, most of which are smaller than 30 kDa and more diverse than other inducible Hsps in the sequence, except a conserved α-crystallin domain of ∼90 amino acids ( Sørensen et al. 2003 , Stahl et al. 2007 , Lü and Wan 2011 , Hanazono et al. 2012 ). In insects, Hsps are known to confer temperature tolerance. In addition to this function, Hsps are also integral to normal cell growth and development, and the expression patterns of Hsps are in general consistent with enhanced stress such as cold winter temperatures encountered by insects ( Feder and Hofmann 1999 , Morrow and Tanguay 2012 ). Considering the critical role of Hsps in response to irreversible stress conditions in many insects, it is presumed that Hsps can also be involved in the temperature adaptation of *E. pela* . To explore this possibility, the full-length cDNA sequences of four *hsp* s ( *hsp21.5* , *hsp21.7* , *hsp70* , and *hsc70* ) were selected from the transcriptome of *E. pela* and cloned. In addition, the *hsc70* genomic sequence was also characterized. Transcriptional expression patterns of the four *E. pela hsp* s under temperature stress and during various developmental stages were examined by quantitative real-time polymerase chain reaction (qRT-PCR). Our results of this study will promote greater understanding of the basis for the adaptation and population variations within *E. pela* at a molecular level.

## Materials and Methods

### 

#### Insects

*E. pela* samples were collected from the Chinese private *Ligustrum lucidum* in the Research Institute of Resources Insects.

Samples of *E. pela* were collected at the following developmental stages: a mixture of male and female eggs, first- and second-stage larvae of male and female, male pupae, male adults and female adults at early- (FA1), middle- (FA2), and late stages (FA3).

To induce heat stress, female adults and male second-stage larvae, which have the longest developmental stages for each sex, were exposed to the following temperatures for 1 h: 45°C, 42°C, 39°C, 36°C, and 33°C. To simulate cold stress, temperatures were set at −20°C, −15°C, −10°C, −5°C, 0°C, and 5°C. Female adults and male second-stage larvae maintained at 25°C were used as controls. Immediately after treatment, all samples were separately homogenized in TRIzol reagent and then stored at −70°C until RNA extraction. Eggs were used as a control group. Three replicates were prepared for each experimental group.

#### cDNA Synthesis and RACE

Total RNA from adult females was isolated using TRIzol reagent (Invitrogen, California, USA) according to the manufacturer’s instructions and used as template to generate cDNA using SMARTer RACE (Rapid Amplification of cDNA Ends) cDNA Amplification kit (Clontech, CA). Specific primers used for RACE were designed based on *hsp* sequences identified in the *E. pela* transcriptome ( Yang et al. 2012 ). Primer sequences for *hsp21.5* , *hsp21.7* , *hsp70* , and *hsc70* used in 5′- and 3′-RACE are listed in Table 1 . PCR products from 5′- and 3′-RACE reactions were cloned into the pGEM T-Easy vector (Promega, Shanghai, China) and sequenced. Assembly of all sequences of cDNA products was done by using DNAMAN software (version 6.0, Lynnon Corporation; http://www.lynnon.com/pc/pcmain_new.html ).

**Table 1.** Primers used in the study

Gene		5′- to 3′-Primer sequences	
Primers used in RACE			
*hsp20.5*	5′-1		GGGCCTCAACTGTCAATACACCATCT
	5′-2		TTGTCCACCGTCTTGACCACGATTTCCT
	3′-1		CGACTAGCACCTCAAATACCGT
	3′-2		TGAAGCTGCGATTCGACGTGAG
*hsp20.7*	5′-1		TAGCTGAGGTATTCGTGCGAGTGAT
	5′-2		CCTCGATGACCAAATAATCGTCCACAAT
	3′-1		GTTCAGCAATTCAAACCCGAAGAAAT
	3′-2		CGCCAATTCACACGTCGTTACAGATT
*hsp70*	5′-1		CGATTTCCTCCTTTGACAGTCTTCCT
	5′-2		GTCGATCTAAACAAGTCCGAGCACAAT
	3′-1		GATTCAAGGATGAGGACGAGAAACAG
	3′-2		GAACACGTTGGCCGAGAAAGATGAGTAT
*hsc70*	5′-1		TCCAACCGGCGAATCGTTTCGTTACAT
	5′-2		GTCTTTGCTAAGTCTACCCTTGTCGTT
	3′-1		GCCAACGGTATTTTGAATGTGAGT
	3′-2		CGCCGGTTGGACTCGAATCAATTAGCT
Primers used in genomic DNA clone			
*hsc70*	5′		ACGGTGAAACTGAAACTGAAG
	3′		CTCCAGCGACATTGTATTATTC
Primers used in RT-PCR			
*hsp20.5*	F		GATAACGAGTTCAGCAGCAT
	R		TGGATACGGTATTTGAGGTG
*hsp20.7*	F		GCCAATTCACACGTCGTTAC
	R		CGGAGCAACAATCGATAGGA
*hsp70*	F		CTCGTGGTATCCCTCAAATC
	R		CCTCCTTTGACAGTCTTCCT
*hsc70*	F		ACGGTATTTTGAATGTGAGT
	R		TCTTCAACGGTGCTCTTCAT
*β-actin*	F		CCACGAGACGACCTACAAT
	R		CGATCCATACGGAGTACT

#### Amplification of Genomic DNA

Genomic DNA was isolated from adult female *E. pela* using DNeasy Blood & Tissue kit (Qiagen, Dusseldorf, Germany). Primers ( Table 1 ) located in the 5′- and 3′-untranslated regions (UTRs) of *hsc70* cDNA were used to amplify *hsc70* genomic DNA. Thermal cycling conditions were as follows: 94°C for 4 min followed by 31 cycles of 94°C for 1 min, 56°C for 1 min, and 72°C for 5 min, with a final extension at 72°C for 10 min.

#### Sequence Analysis

Amino acid sequences of insect Hsps were downloaded from NCBI after BLAST search ( http://www.ncbi.nlm.cih.gov/blast/Blast.cgi ). Sequence alignments were conducted by using DNAMAN. Open reading frames (ORFs) were identified using the ORF Finder ( http://www.ncbi.nlm.cih.gov/gorf/gorf.html ). Molecular mass of the predicted proteins was calculated using the Compute pI/Mw program available online ( http://www.expasy.ch/tools/pi_tool.html ), and *E. pela* Hsps were named according to their predicted molecular mass.

A neighbor-joining tree was constructed using the Poisson-correction distance method ( Nei and Kumar 2000 ) based on the full-length amino acid sequences. Alignment of the selected amino acid sequences was first performed using the CLUSTALX1.83 software ( Thompson et al. 1997 ) and then imported to MEGA5.0. Phylogenetic trees were generated using the neighbor-joining method and bootstrapped with 1,000 iterations to evaluate the branch strength of the tree. Bootstrap percentages of 1,000 replicates that were >50% were shown at the nodes ( Tamura et al. 2011 ). sHsp from *Arabidopsis thaliana* and *Glycine max* were used as outgroups.

#### qRT-PCR Analysis

Specific primer pairs were designed based on the sequences of *hsp* s and *β-actin* from *E. pela* ( Table 1 ). qRT-PCR was performed on a CFX96 Real-Time PCR Detection System in 20 µl reactions that included 10 µl SsoFast EvaGreen Supermix (Bio-Rad, CA), 1 µl of each of the forward and reverse primers (20 µM), and 2 µl of cDNA template. PCR cycling conditions were as follows: initial denaturation at 95°C for 3 min, followed by 30 cycles at 95°C for 10 s and 55°C for 20 s. Melting curves were generated to ensure specificity of the PCR products with temperatures from 65 to 95°C, increased in 0.5°C, 5 s increments. Relative gene expression levels of each *hsp* were calculated using the 2 ^−^^ΔΔCt^ method ( Livak and Schmittgen 2001 ) with *β-actin* as an internal standard for normalization. The least significant difference test at *P* < 0.01 level was performed with the DPS statistical program to analyze gene expression variations ( Tang and Feng 2010 ).

## Results

### 

#### Sequence Analysis of *ep-hsp21.5* and *ep-hsp21.7*

**Fig. 1.**
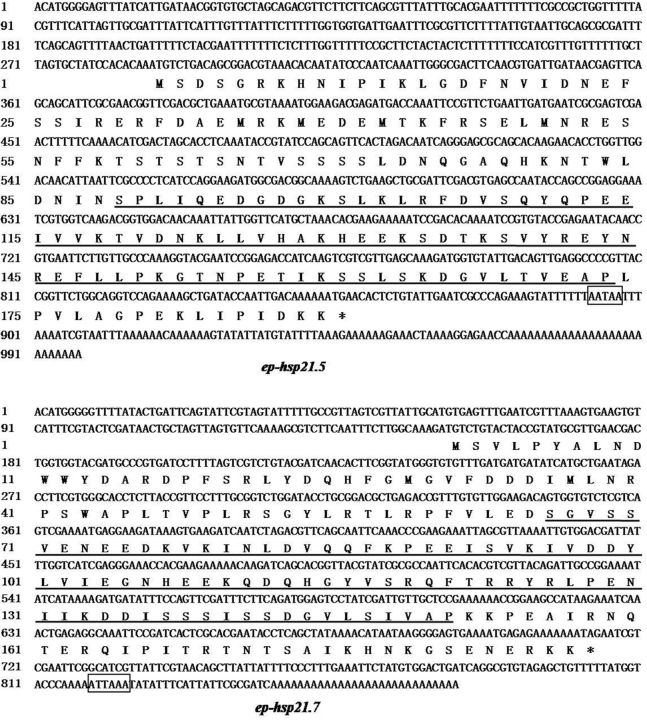
Complete nucleotide and deduced amino acid sequences of *ep-hsp21.5* and *ep-hsp21.7* cloned from *E. pela* . Translation termination codon is marked with an asterisk, putative polyadenylation signal is boxed, and the alpha-crystallin domain is underlined.

The full-length sequences of *ep-hsp21.5* and *ep-hsp21.7* (GenBank accession numbers: KC161298 and KC161299, respectively) were 997 and 873 bp in length and contained 570 and 564 bp ORFs, respectively. The ORF of *ep-hsp21.5* encoded a putative protein of 189 amino acids with a calculated molecular mass of 21.5 kDa, and the ORF of *ep-hsp21.7* encoded a deduced protein of 187 amino acids with a molecular mass of 21.7 kDa. *ep-hsp21.5* contained a 287-bp 5′-UTR and 140-bp 3′-UTR, whereas *ep-hsp21.7* had a 150-bp 5′-UTR and 159-bp 3′-UTR. The termination codon (TGA) was located at 855 and 712 bp, followed by the polyadenylation signal AATAA ( Huang et al. 2008 ) in *ep-hsp21.5* and ATTAA ( Beaudoing et al. 2000 ) in *ep-hsp21.7* ( Fig. 1 ). Blastp comparisons of the deduced ep-Hsp21.5 amino acid sequence shared a 77% identity with sHsp of *Spodoptera litura* (ADK55519), and ep-Hsp21.7 shared a 55% identity with the putative sHsp from *Maconellicoccus hirsutus* (ABM55532). Multiple sequence alignment showed that all sequences contained the conserved α-crystallin domain ( Fig. 1 ), indicating that the isolated *ep-hsp21.5* and *ep-hsp21.7* sequences were *shsp* s.

**Fig. 2.**
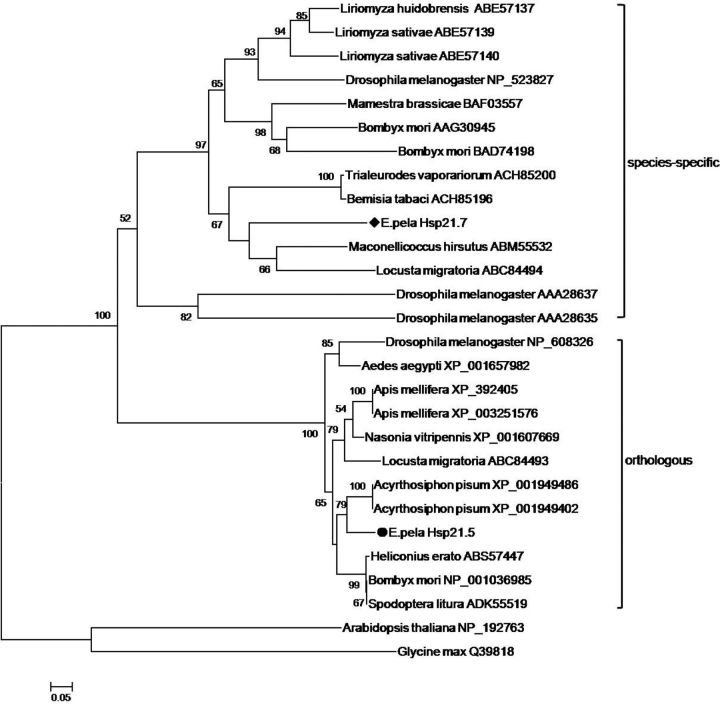
Phylogenetic analysis of Hsp20 sequences. Some orthologous sequences used in this analysis are from Li et al. (2009) . Sequence labels are denoted by the species names and GenBank accession numbers. Black diamond indicates the sequence of ep-Hsp21.7, and black round indicates the sequence of ep-Hsp21.5.

The phylogenetic tree analysis showed that ep-Hsp21.7 was clustered into the species-specific sHsp group, whereas ep-Hsp21.5 was clustered into the orthologous sHsp group ( Fig. 2 ).

#### Sequence Analysis of *ep-hsp70* and *ep-hsc70*

**Fig. 3.**
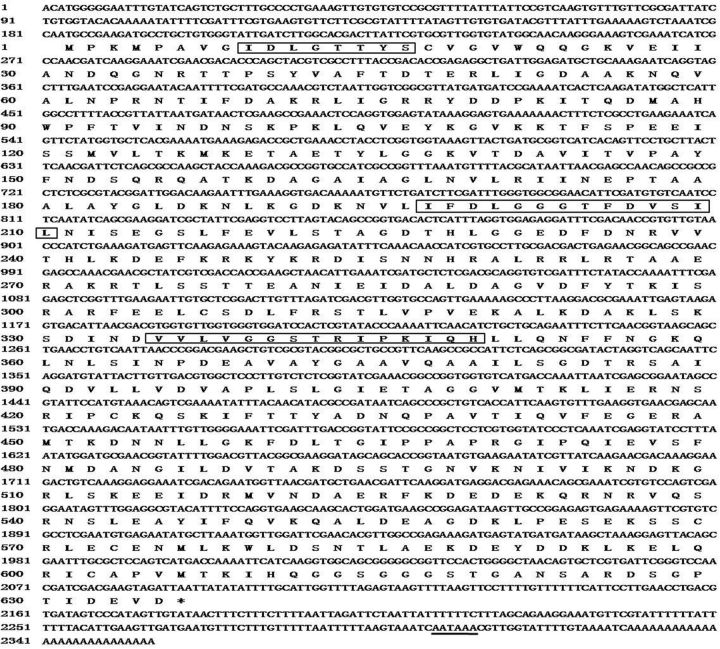
The complete nucleotide and deduced amino acid sequences of *ep-hsp70* cloned from *E. pela* . The three characteristic signature sequences of the Hsp70 family are boxed. Translation termination codon is marked with an asterisk, and the putative polyadenylation signal is underlined.

**Fig. 4.**
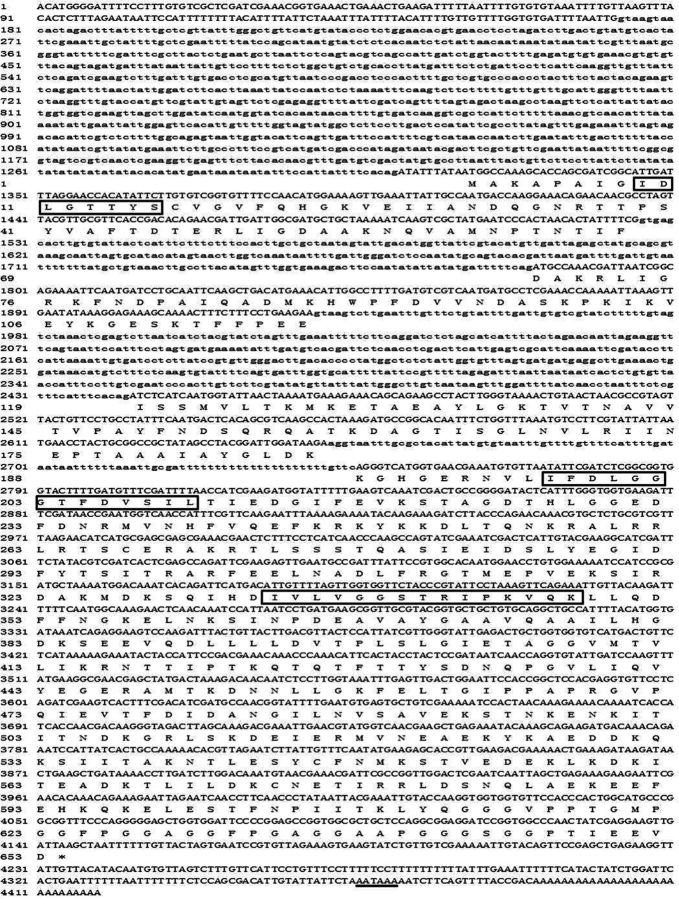
The nucleotide and deduced amino acid sequences of *ep-hsc70* cloned from *E. pela* . The three characteristic signature sequences of the Hsp70 family are boxed. Translation termination codon is marked with an asterisk, and the putative polyadenylation signal is underlined. Nucleotides in lowercase letters represent the intron region.

Full-length cDNA sequences of *ep-hsp70* (GenBank accession number: KC161300) and *ep-hsc70* (GenBank accession number: KC161301) were 2,355 and 2,417 bp in length with 1,908 and 1,962 bp, respectively. *ep-hsp70* encoded a deduced protein of 635 amino acids with a calculated molecular mass of 69.8 kDa. *ep-hsc70* encoded a deduced protein of 653 amino acids with a calculated molecular mass of 71.6 kDa. *ep-hsp70* contained a 182-bp 5′-UTR and 265-bp 3′-UTR, whereas *ep-hsc70* contained a 181-bp 5′-UTR and 274-bp 3′-UTR. The termination codon (TAA) was present at 2,088 ( *hsp70* ) and 2,141 ( *hsc70* ) bp, followed by the polyadenylation signal (AATAAA) and a poly (A) tail. Three characteristic signature sequences of the Hsp70 family were observed in the amino acids sequences of ep-Hsp70 and ep-Hsc70 *,* including IDLGTTYS, IFDLGGGTFDVSIL, and IVLVGGSTRIPKIQK ( Figs. 3 and 4 ). The conserved motif EEVD usually present in the *C* -terminus of cytoplasmic Hsp70 was altered to DEVD in ep-Hsp70. A Blastp search revealed that the deduced amino acid sequence of ep-Hsp70 shared an 81% identity with *Bemicia tabaci* Hsp70 (ADO14473) and ep-Hsc70 shared a 92% identity with *Nilaparvata lugens* Hsc70 (ADE34170).

**Fig. 5.**
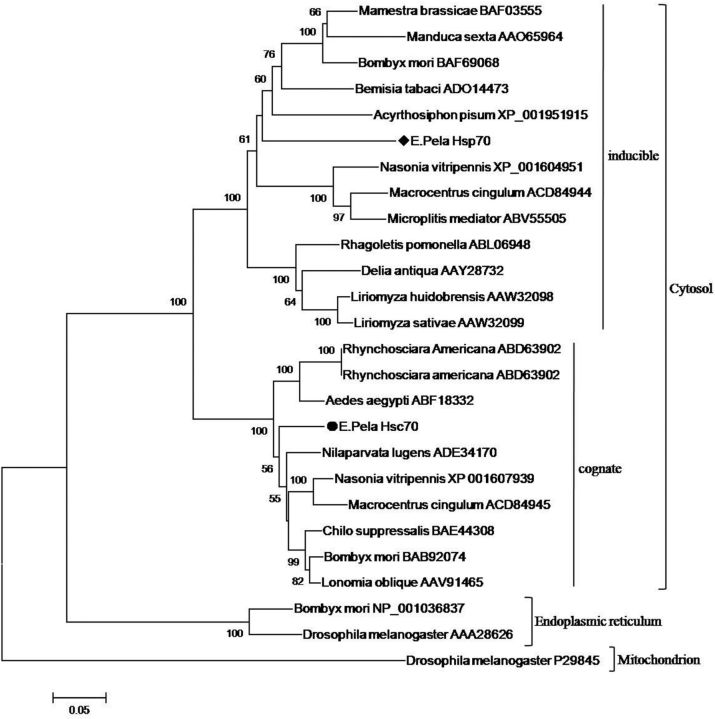
Phylogenetic analysis of Hsp70 sequences. Some sequences used in this analysis are previously reported by other groups ( Huang et al. 2008 , Xu et al. 2010 , Aruda et al. 2011 ). Sequence labels are denoted by the species names and GenBank accession numbers. Black diamond indicates the sequence of ep-Hsp70, and black round indicates the sequence of ep-Hsc70.

The phylogenetic tree analysis of Hsp70 showed that ep-Hsp70 was clustered together with inducible Hsp70, whereas ep-Hsc70 was clustered with cognate Hsc70 of other species. However, both ep-Hsp70 and ep-Hsc70 were clustered with cytoplasmic Hsp70 ( Fig. 5 ).

The genomic DNA sequence of *hsc70* (GenBank accession number: KC161301) had four introns, which were 1,139, 255, 514, and 94 bp in length. The first intron was the longest from 173 to 1,311 bp and located in the 5′-UTR, whereas the remaining three introns were located in the coding regions, from 1,526 to 1,780 bp, 1,930 to 2,443 bp, and 2,652 to 2,745 bp, respectively ( Fig. 4 ). The intron–exon junctions of the first three introns followed the GT-AG rule, whereas the fourth intron–exon junction was AG-TC.

#### Expression of the *ep-hsps* in Female and Male *E. pela* Under Temperature Stress

**Fig. 6.**
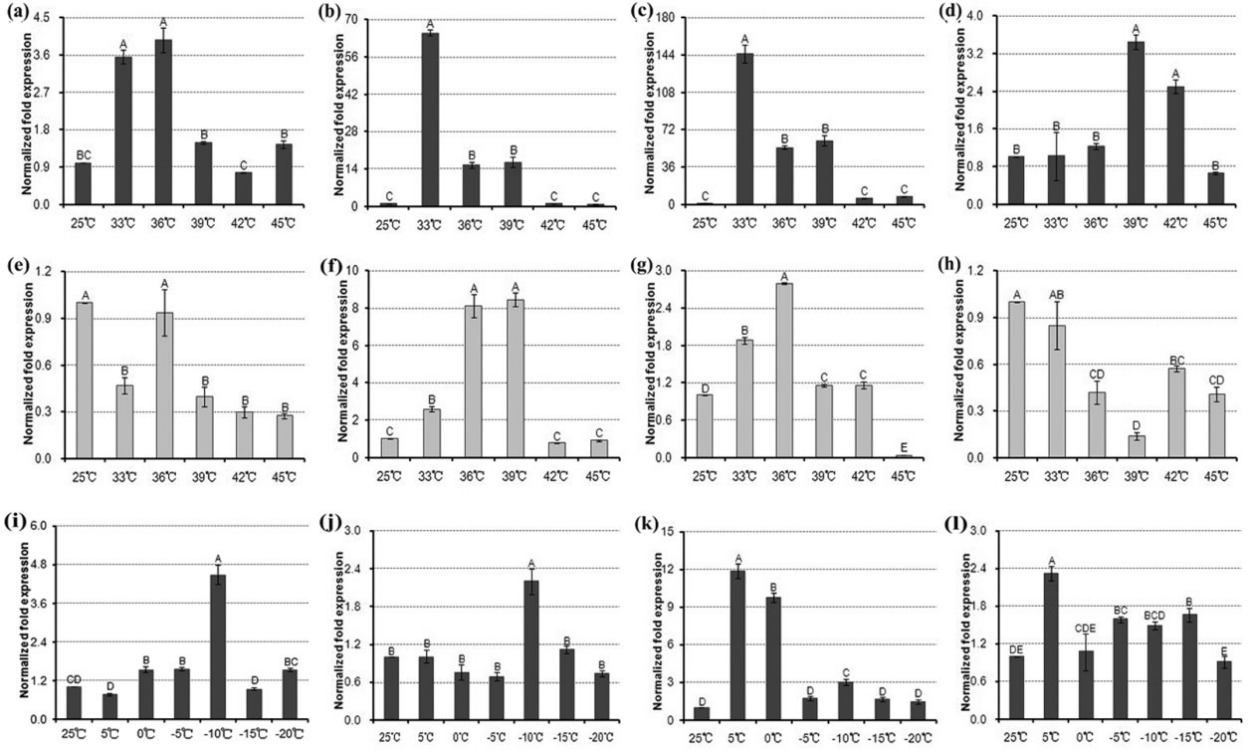
Relative mRNA expression levels of each *hsp* under temperature stresses. *hsp21.5* (a), *hsp21.7* (b), *hsp70* (c), *hsc70* (d) in female *E. pela* under heat shock, *hsp21.5* (e), *hsp21.7* (f), *hsp70* (g), *hsc70* (h) in male *E. pela* under heat shock, *hsp21.5* (i), *hsp21.7* (j), *hsp70* (k), *hsc70* (l) in female *E. pela* under cold shock. The values are mean ± SE of three replicates for each of the treatments; 25°C is the control. Letters above columns indicate levels of difference significance at *P* < 0.01. The same letters are not significantly different, *P* > 0.01.

qRT-PCR revealed that in females, the mRNA expression levels of all four *hsp* s were correlated with heat stress. In adult females at 33°C, the expression of *hsp21.7* and *hsp70* were drastically upregulated by 65.02 and 145.31 folds, respectively, over the control expression at 25°C ( Fig. 6 B and C). mRNA expression of *hsp21.5* and *hsc70* was also significantly upregulated, when compared with the control ( Fig. 6 A and D).

In male second-stage larvae, mRNA levels of *hsp21.7* increased when the temperatures were between 33 and 39°C and peaked at 39°C, 8.45-fold over the control at 25°C ( Fig. 6 F). *hsp70* was also upregulated in males under heat stress ( Fig. 6 G), whereas expression levels of *hsp21.5* and *hsc70* mRNA were downregulated by the heat stress ( Fig. 6 E and H).

All four *ep-hsp* s were significantly induced by cold stress in female adults. *hsp21.5* was upregulated by the cold treatments with a peak at −10°C, which was a 4.48-fold increase when compared with the control at 25°C, whereas *hsp70* reached a peak at 5°C, 11.88-fold over the control ( Fig. 6 I and K).

#### Expression of *ep-hsps* in Female and Male *E. pela* at Different Developmental Stages

**Fig. 7.**
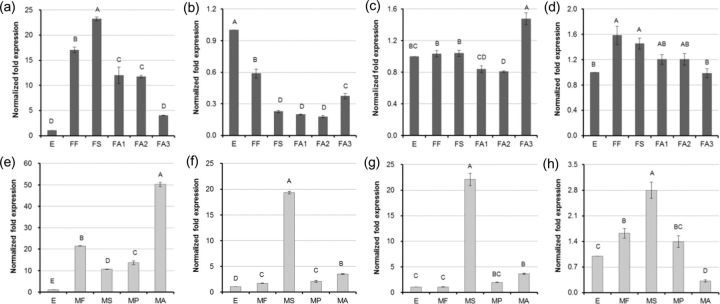
Relative mRNA expression levels of *hsps* at different developmental stages in both female and male *E. pela* . *hsp21.5* (a), *hsp21.7* (b), *hsp70* (c), and *hsc70* (d) in female *E. pela* ; *hsp21.5* (e), *hsp21.7* (f), *hsp70* (g), and *hsc70* (h) in male *E. pela* . E, mixture of female and male eggs; FF, female first-stage larvae; FS, female second-stage larvae; FA1, early female adults; FA2, mid female adults; FA3, late female adults; MF, male first-stage larvae; MS, male second-stage larvae; MP, male pupae; MA, male adults. Eggs are the control. Letters above columns indicate levels of difference significance at *P* < 0.01. The same letters are not significantly different, *P* > 0.01.

In females, the mRNA levels of *hsp21.5* at each of larval, pupal, and adult stages were higher than eggs ( Fig. 7 A). The expression of *hsp21.5* reached a maximum in second-stage larvae with a 23.22-fold increase when compared with eggs. The expression of *hsp21.7* was very low through all stages of the female development ( Fig. 7 B). *hsp70* was upregulated significantly in the late female adults ( Fig. 7 C). *hsc70* was upregulated significantly in the first-stage larvae ( Fig. 7 D).

In males, the mRNA levels of *hsp21.5* in all developmental stages were also remarkably higher than in eggs ( Fig. 7 E). Specifically, the mRNA level of *hsp21.5* was maximal in adults, showing a 50.21-fold increase over the eggs. The mRNA levels of other *hsp* s peaked in second-stage larvae. The mRNA levels of *hsp21.7* and *hsp70* in second-stage larvae were remarkably higher than in other developmental stages, increasing 19.31- and 22.08-fold, respectively, when compared with expression in the eggs ( Fig. 7 F and G). The *hsc70* mRNA peaked at the second larval stage and then decreased in pupae and adult stages ( Fig. 7 H).

## Discussion

In insects, a number of *shsp* s are reported to be involved in temperature adaptation and development. For example, six *shsp* s, which are classified into three groups with diverse functions, are reported in the common cutworm, *S.litura* ( Shen et al. 2011 ), and three *shsp* s are found to be responsible for cold stress and development in the leafminer, *Liriomyza sativa* ( Huang et al. 2009 ). In this study, two *shsp* s ( *hsp21.5* and *hsp21.7* ) were identified and cloned. Phylogenetic analysis showed that *hsp21.5* was classified into the orthologous cluster, and *hsp21.7* was classified into the species-specific cluster ( Li et al. 2009 ). Orthologous *shsp* s, which include *ep-hsp21.5,* have highly conserved sequences, gene structures, and functions, and they have at least one intron in their genes. Expressions of these *shsp* s are usually constitutive but are not inducible in response to environmental stresses. They are involved in basic metabolic processes. On the other hand, *shsp* s, including *ep-hsp21.7* , in the species-specific group are clustered in specific insect order. These intron-less *shsp* s are important in responses to temperature stimuli and display remarkable mRNA expression differences in mRNA expression levels during insect development. Although most insect sHsps identified so far are species specific, many orthologous sHsps have also been indentified in insects, nematodes ( Lillibridge et al. 1996 ), amphibians ( Franck et al. 2004 ), and mammals ( Kampinga et al. 2009 ). However, orthologous and species-specific sHsps may be involved in various functions in different insects ( Shen et al. 2011 ).

In *E. pela* females, *ep-hsp21.5* was upregulated in larval stages, and expression in adults was also significantly higher than expression measured in eggs. Similar upregulation was also observed during male insect development, suggesting a correlation between *ep-hsp21.5* and the developmental regulation of female and male *E. pela* . In male larvae, *ep-hsp21.5* was not sensitive to heat stimuli, whereas, in female adults, *ep-hsp21.5* was upregulated significantly by the heat- and cold-stress treatments, which may explain why *E. pela* female adults can persist in −30°C temperatures in Changchun, China. The expression pattern of *ep-hsp21.5* in larval stage was consistent with the expression of orthologous *shsp* s in most insects.

In contrast, *ep-hsp21.7* was more sensitive to the heat or cold stimulus than *hsp21.5,* indicating that this gene may play more important role in temperature-stress tolerance than *hsp21.5* . Our data were also consistent with studies in *Bombyx mori* , in which heat shock triggered a massive increase of six species-specific *shsp* s but not the orthologous *hsp21.4* ( Sakano et al. 2006 ). However, *ep-hsp21.7* was upregulated drastically at the second larval stage, when the wax secretion in males is at the maximum. Studies to investigate the ecological adaptability of *E. pela* and analyses of climatic factors that affect wax secretion have suggested that wax secretion is an ecological strategy and a protective response to severe environmental conditions ( Chen et al. 2007b ). Thus, *ep-hsp21.7* was presumed to be correlated with the protective response to environment stress by secreting wax.

The members of the Hsp70 family are found to be present in the cytoplasm, mitochondria, endoplasmic reticulum, and chloroplast depending on the sequences specific to the each cellular organelle. For example, cytoplasmic Hsp70 has the specific motif EEVD. Phylogenetic analysis revealed the two ep-Hsp70 of *E. pela* were clustered to the group of cytoplasm Hsp70 by their cellular localization rather than insect orders. The expression of *ep-hsp70* was inducible and the expression of *ep-hsc70* was constitutive. Usually, *hsc70* genes have introns, whereas most *hsp70* genes have no introns, which may facilitate their rapid transcription during stress because no mRNA splicing is needed. In this study, *ep-hsp70* was typically induced by temperature shock, especially in adults. It was also drastically upregulated in larval stage of male second-stage larvae. However, significantly high expression level was detected in both female adults (FA3) and male adults, indicating that this gene may be correlated with the development of adults.

Generally, when compared with *hsp70* , *hsc70* is constitutively expressed in many insects and does not significantly change under conditions of stress ( Shim et al. 2006 , Colinet et al. 2009 , Koštál and Tollarová-Borovanská 2009 ). In *E. pela* , *ep-hsc70* was upregulated during the development of females and males, indicating that it may be associated with development. In addition, *ep-hsc70* did not respond to heat shock at the larval stage (male second-stage larvae), which was consistent with many reports about *hsc70* . However, *ep-hsc70* was sensitive to heat or cold stimulus in adult stages (female adults). The results of this study showed that *ep-hsc70* was responsible to the heat or cold-stress treatments in female adults, suggesting that this gene may be correlated with temperature–stress tolerance of the female insect in extreme temperatures. The special expression pattern of *hsc70* was also reported in *Pteromalus puparum* ( Wang et al. 2008 ) and *Sarcophaga crassipalpis* ( Rinehart et al. 2000 ).

As a chaperone molecule, *hsp* s have been thought to be involved in insect developmental processes. Specifically, they play vital roles in cell differentiation, cell cycle regulation, and embryonic development ( Mahroof et al. 2005 , Tachibana et al. 2005 , de Andrade et al. 2009 , Zhang and Denlinger 2010 ). *E. pela* has intriguing biological characteristics, but not much is known about the expression patterns of *hsp* s at extreme temperatures. This study revealed that *ep-hsp21.5* , *ep-hsp21.7* , *ep-hsp70,* and *ep-hsc70* may be correlated with heat or cold-stress tolerance in female adults, whereas *ep-hsp21.7* and *ep-hsp70* could be correlated with heat stress in male larvae. The four *ep-hsp* s may be involved in the developmental regulation of males, and *ep-hsp21.5* , *ep-hsp70* , and *ep-hsc70* may be critical for developmental regulation of females. However, the physiological functions of these *ep-hsp* s during development of *E. pela* need further investigation.
